# Supplementary figures and images for: Clinical performance of zirconium implants compared to titanium implants: a systematic review and meta-analysis of randomized controlled trials
Source: PeerJ. 2023 Mar 17;11:e15010. doi: 10.7717/peerj.15010 (PMC10026713; doi:10.7717/peerj.15010)

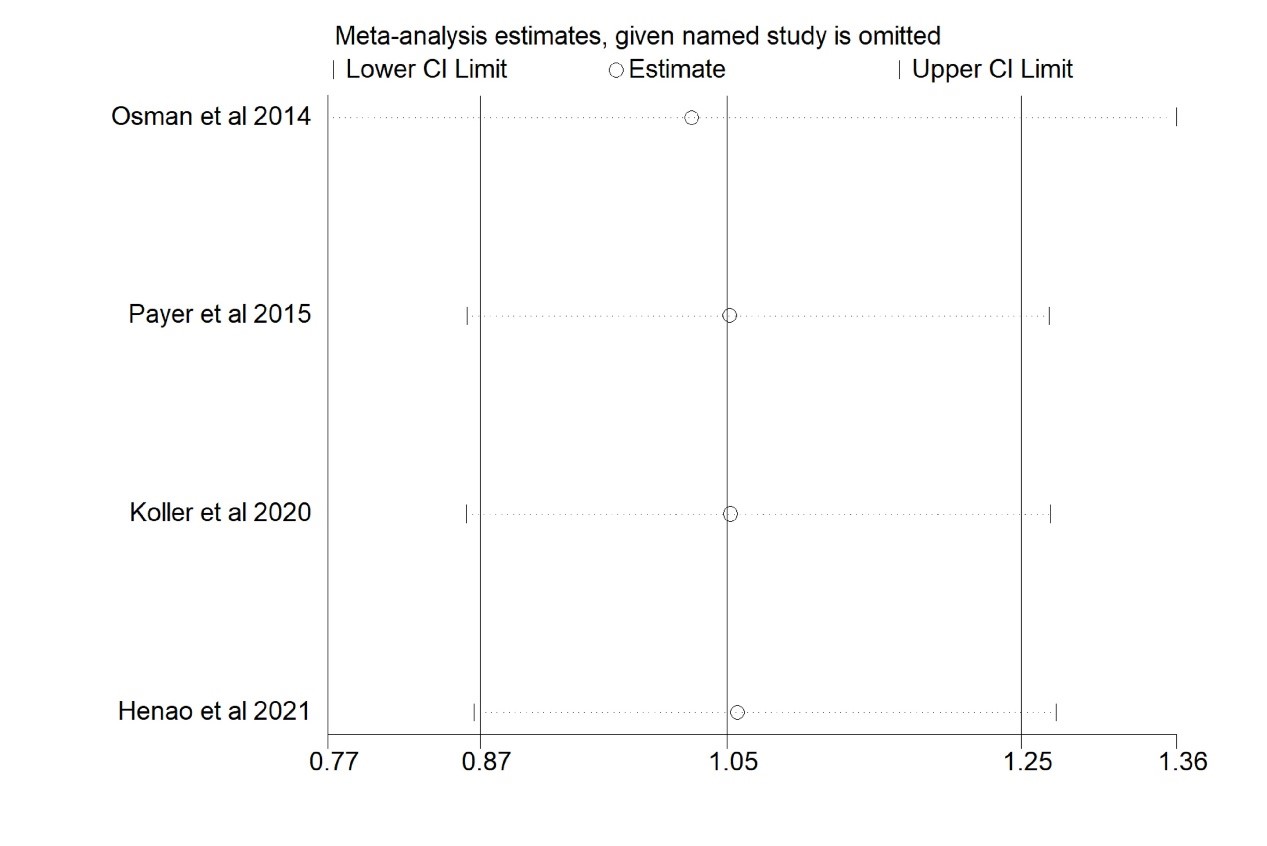

Supplement: Figure S1 [file peerj-11-15010-s002.jpg]

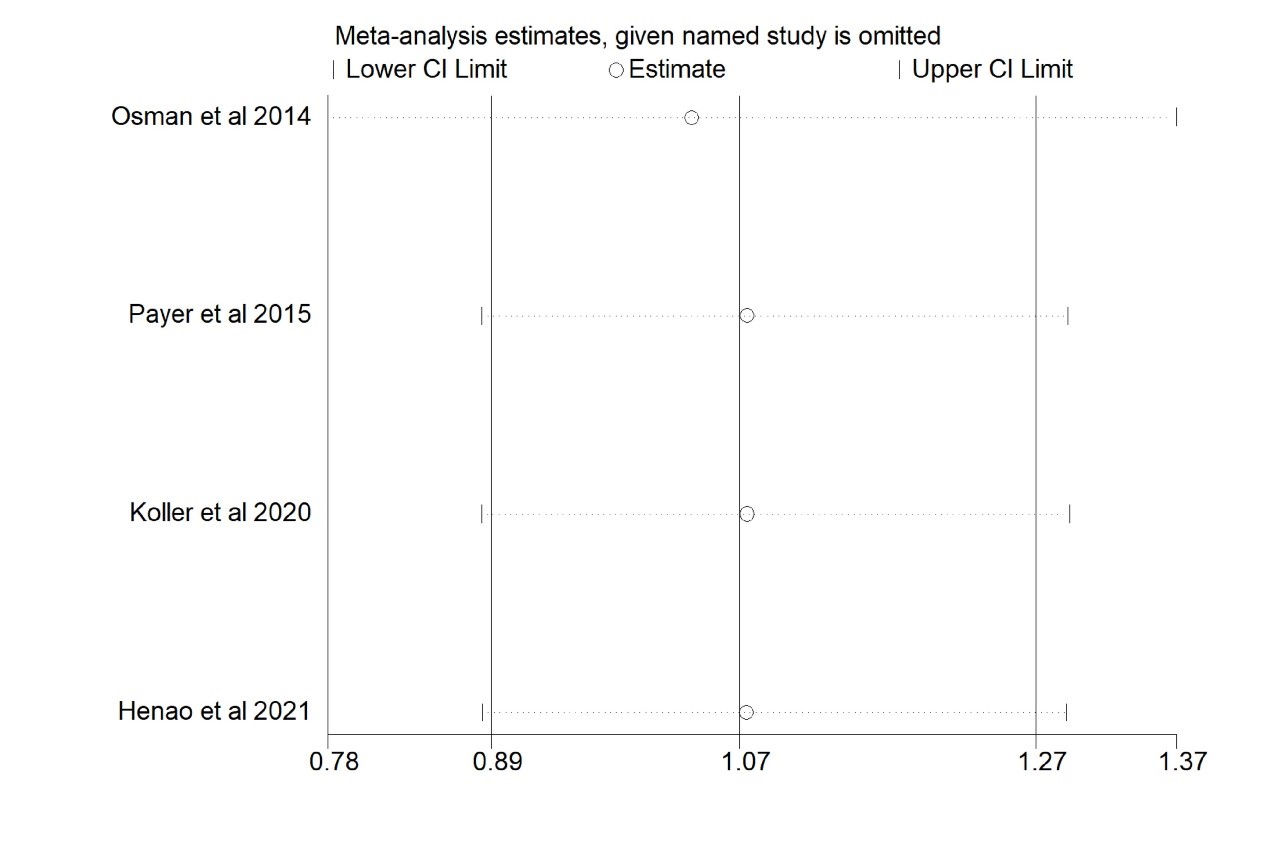

Supplement: Figure S2 [file peerj-11-15010-s003.jpg]

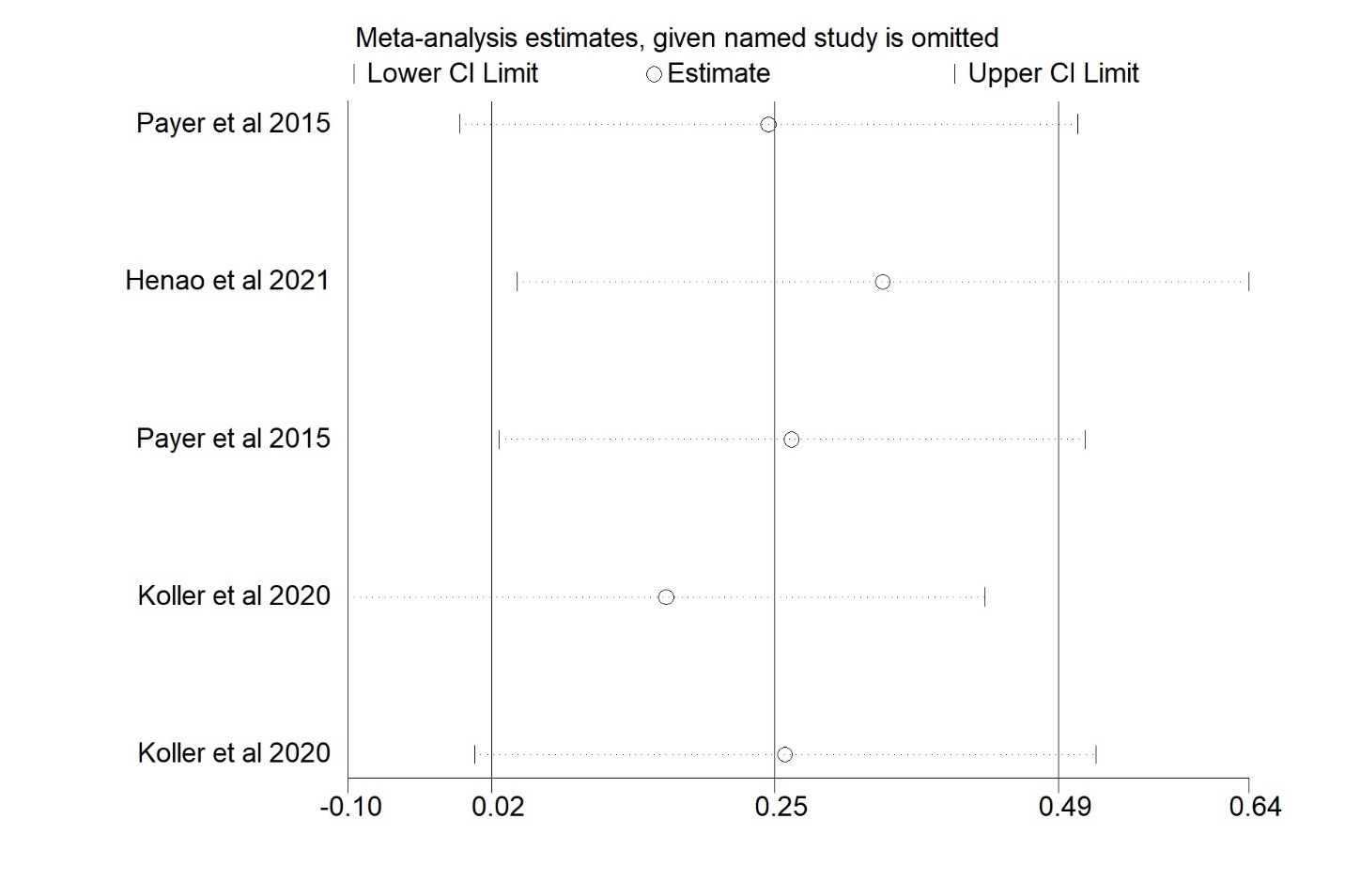

Supplement: Figure S3 [file peerj-11-15010-s004.jpg]

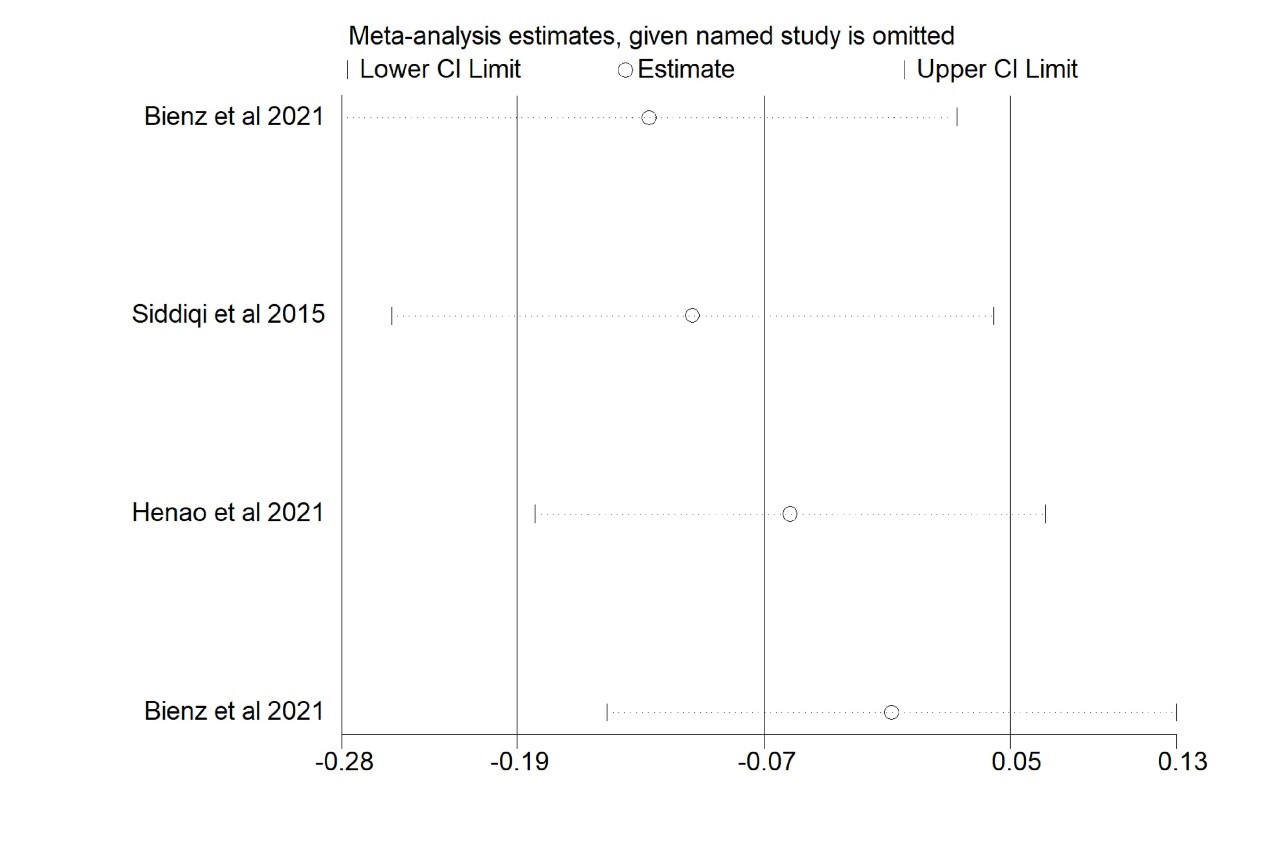

Supplement: Figure S4 [file peerj-11-15010-s005.jpg]
