# Supplementary material for: Clinical performance of zirconium implants compared to titanium implants: a systematic review and meta-analysis of randomized controlled trials
Source: PeerJ. 2023 Mar 17;11:e15010. doi: 10.7717/peerj.15010 (PMC10026713; doi:10.7717/peerj.15010)
Supplement: Table S1 [file peerj-11-15010-s006.docx]

**Table S1:** The string of specific search strategies of databases, including Cochrane Central Register of Controlled Trials (CENTRAL), MEDLINE via OVID, EMBASE, Web of Science.

| Electronic database and search strategy | |
| --- | --- |
| Cochrane Central Register of Controlled Trials (CENTRAL) | #1 MeSH descriptor: [Dental Implants] explode all trees |
|  | #2 MeSH descriptor: [Zirconium] explode all trees |
|  | #3 #1 AND #2 |
| MEDLINE via OVID | #1 (dental implants [Mesh] OR implant.m.p.) |
|  | #2 (Zr oxide [Mesh] OR Ceramics [Mesh] OR Zirconium.m.p .OR Zirconium.m.p) |
|  | #3 #1 AND #2 |
| EMBASE | #1 'tooth implant'/exp |
|  | #2 'implant, dental':ab,ti OR 'implants, dental':ab,ti OR 'dental implant':ab,ti |
|  | #3 'zirconium oxide'/exp |
|  | #4 'zirconium dioxide':ab,ti OR zirconia:ab,ti |
|  | #5 'ceramics'/exp |
|  | #6 ceramic:ab,ti OR 'ceramic industry':ab,ti OR 'ceramic prosthesis':ab,ti |
|  | #7 #1 OR #2 |
|  | #8 #3 OR #4 OR #5 OR #6 |
|  | #9 #7 AND #8 |
| Web of Science | #1 TS = (Dental Implants) OR TS=(Implant, Dental) OR TS=(Implants, Dental) OR TS=(Dental Implant) |
|  | #2 TS = (zirconium oxide) OR TS = (zirconium) OR TS = (ceramics) OR TS = (yttria stabilized tetragonal zirconia) |
|  | #3 #1 AND #2  Refined by: DOCUMENT TYPES: (ARTICLE OR CLINICAL TRIAL) Timespan: 1975 to March 2022. Databases: WOS, DIIDW, INSPEC, KJD, MEDLINE, RSCI, SCIELO.  Search language = Auto |

The search time limit was from the inception of the database to August 2022.
