# Supplementary material for: Clinical performance of zirconium implants compared to titanium implants: a systematic review and meta-analysis of randomized controlled trials
Source: PeerJ. 2023 Mar 17;11:e15010. doi: 10.7717/peerj.15010 (PMC10026713; doi:10.7717/peerj.15010)
Supplement: Table S2 [file peerj-11-15010-s007.docx]

**Table S2:**

Summary of findings and certainty of evidence assessed by GRADE.

| Outcome  No. of implants  (studies) | Relative effect (95% CI) | Certainty |
| --- | --- | --- |
| Survival Rate (Survival Rate)  assessed with: %  No. of implants: 221  (4 RCTs) | RR 0.91 (0.82 to 1.02) | ⨁⨁◯◯  Low^a,b,c^ |
| Success Rate (Success Rate)  assessed with: %  No. of implants: 221  (4 RCTs) | RR 0.87 (0.78 to 0.98) | ⨁⨁⨁◯  Moderate^a^ |
| Marginal bone loss (MBL)  assessed with: mm  No. of implants: 61  (3 RCTs) | MD 0.25 (0.02 to 0.49) | ⨁◯◯◯  Very low^a,d,e^ |
| Pocket probing depth (PPD)  assessed with: mm  No. of implants: 208  (4 RCTs) | MD -0.07 (-0.19 to 0.05) | ⨁⨁◯◯  Low^a,b^ |
| Bleeding on probing (BOP)  assessed with: %  No. of implants: 71  (3 RCTs) | not pooled | ⨁⨁◯◯  Low^b,d^ |
| Plaque index (PI)  assessed with: %  No. of implants: 219  (4 RCTs) | not pooled | ⨁◯◯◯  Very low^a,b,e^ |
| Pink esthetic score (PES)  No. of implants: 73  (3 RCTs) | not pooled | ⨁⨁◯◯  Low^d,e^ |

Footnotes:

a. Downgraded one level due to study limitations: high withdrawal rate lowered the confidence in the estimate of effect in one or more studies; b. Downgraded one level due to study limitations: method of randomization or allocation not described in one or more studies; c. Downgraded one level due to imprecison: significant difference between groups in baseline data; d. Downgraded one level due to imprecision: low number of participants; e. Downgraded one level due to inconsistency of results.
