# Supplementary material for: Clinical performance of zirconium implants compared to titanium implants: a systematic review and meta-analysis of randomized controlled trials
Source: PeerJ. 2023 Mar 17;11:e15010. doi: 10.7717/peerj.15010 (PMC10026713; doi:10.7717/peerj.15010)
Supplement: Table S3 [file peerj-11-15010-s008.docx]

**Table S3:**

Success rate and reasons of prothesis failure in Zirconium implants.

| Study | Total | Success | Success rate | Reasons of prothesis failure | Type of prostheses | Implant | Drop out | Anterior/  Posterior | Maxilla/ Mandible |
| --- | --- | --- | --- | --- | --- | --- | --- | --- | --- |
| 1-year follow-up | | | | | |  |  |  |  |
| Henao et al 2021(Ruiz Henao et al. 2021) | 16 | 15 | 93.75% | biological complication (peri-implant disease, n = 1) | Monolithic | ZLA Ceramic monotype implant (Straumann) | 0 | 16/0 | NS |
| Koller et al 2020(Koller et al. 2020) | 16 | 15 | 93.75% | technical complication (crown delivery, n = 1) | Monolithic | yttria-stabilized zirconium (Ziterion) | 0 | 3/13 | 3/13 |
| Osman et al 2014(Osman et al. 2014b) | 73 | 49 | 67.12% | technical complication (failed to osseointegrate, n = 18; implants fractured, n = 3; deep placement, n = 3) | Overdentures | Zirconium (Southern Implants) | 11 | 36/48 | 48/36 |
| Cionca et al 2015(Cionca et al. 2015) | 46 | 41 | 89.13% | technical complication (aseptic loosening, n = 5) | Monolithic | two-piece zirconia implants (ZERAMEXâ T Implant System) | 3 | 1/48 | 24/25 |
| Cannizzaro et al 2010(Cannizzaro et al. 2010) | 40 | 32 | 80.00% | biological complication (peri-implant hypertrophic soft tissues, n = 1);  technical complication (crowns fractured, n = 1; decemented crown, n = 1; implants failed, n = 5) | Monolithic | one-piece Z-Look3 zirconia implants (Z-Systems, Oensingen) | 0 | 18/22 | 29/11 |
| Spies et al 2017(Spies et al. 2017) | 24 | 23 | 95.83% | technical complication (occlusal roughness, n = 1) | Monolithic | one-piece zirconia implants (Metoxit AG) | 0 | 4/20 | 12/12 |
| Kniha et al 2017(Kniha et al. 2018) | 105 | 100 | 95.4% | NA | monotype | one-piece zirconia implants (Institut Straumann AG) | 12 | 64/41 (available at end of follow-up) | 82/23 |
| Kohal et al 2012(Kohal et al. 2012) | 62 | 30 | 48.39% | technical complications (implant failed, n=3)  biological complication (bone loss＞3mm, n=27) | Monolithic | yttria-stabilized zirconia (Nobel Biocare) | 4 | 6/50 (radiographs available) | 14/42 |
| Spies et al 2015(Spies et al. 2015) | 62 | 54 | 87.10% | technical complications (chipping of veneering, n = 8) | Veneered | zirconia implants (Biocare®) | 4 | 6/60 | NS |
| Spies et al 2019(Spies et al. 2019) | 44 | 43 | 97.73% | technical complication (chipping of veneering, n = 1) | Veneered | one‐piece yttria‐stabilized zirconia implants (VITA Zahnfabrik) | 0 | 0/44 | 18/26 |
| Spies et al 2015(Spies et al. 2015) | 54 | 36 | 66.67% | technical complications (chipping of veneering, n = 18) | FDP | zirconia implants (Biocare®) | 2 | 6/50 | NS |
| Spies et al 2018(Spies et al. 2018) | 24 | 22 | 91.67% | technical complications (occlusal roughness, n = 2) | FDP | One-piece ceramic implants (Metoxit AG) | 2 | 0/26 | NS |
| Spies et al 2019(Spies et al. 2019) | 20 | 20 | 100.00% | - | FDP | one‐piece yttria‐stabilized zirconia implants (VITA Zahnfabrik) | 0 | NS | NS |
| Total | 586 | 480 | 81.91% | - | - |  |  |  |  |
| 2-year follow-up | | | | | |  |  |  |  |
| Michael Payer et al 2015(Payer et al. 2015) | 16 | 15 | 93.75% | technical complication (crown delivery, n = 1) | Monolithic | yttria-stabilized zirconium (Ziterion) | 0 | 3/13 | 3/13 |
| Koller et al 2020(Koller et al. 2020) | 16 | 15 | 93.75% | technical complication (crown delivery, n = 1) | Monolithic | yttria-stabilized zirconium (Ziterion) | 0 | 3/13 | 3/13 |
| Spies et al 2017(Spies et al. 2017) | 23 | 22 | 95.65% | technical complication (occlusal roughness, n = 1) | Monolithic | one-piece zirconia implants (Metoxit AG) | 1 | 4/20 | 12/12 |
| Becker et al 2017(Becker et al. 2017) | 68 | 47 | 69.12% | biological complication (peri-implantitis, n = 18);  technical complications (implant lost, n = 2; implant fracture, n = 1) | Monolithic | zirconium implant system (Zircon Vision GmbH) | 0 | 0/68 | 18/50 |
| Spies et al 2019(Spies et al. 2019) | 42 | 39 | 92.86% | technical complications (chipping of veneering, n = 1; marginal integrity, n = 1; contour, n = 1) | Veneered | one‐piece yttria‐stabilized zirconia implants (VITA Zahnfabrik) | 2 | 0/44 | 18/26 |
| Spies et al 2015(Spies et al. 2015) | 62 | 50 | 80.65% | technical complication (chipping complications, n = 12) | Veneered | zirconia implants (Biocare®) | 4 | 6/60 | NS |
| Spies et al 2015(Spies et al. 2015) | 50 | 23 | 46.00% | technical complication (hipping complications, n = 27) | FDP | zirconia implants (Biocare®) | 6 | 6/50 | NS |
| Spies et al 2018(Spies et al. 2018) | 24 | 18 | 75.00% | technical complications (occlusal roughness, n = 6) | FDP | One-piece ceramic implants (Metoxit AG) | 2 | 0/26 | NS |
| Total | 301 | 229 | 76.08% | - | - |  |  |  |  |
| 5-year follow-up | | | | | |  |  |  |  |
| Koller et al 2020(Koller et al. 2020) | 16 | 14 | 87.50% | technical complications (crown delivery, n = 2) | Monolithic | yttria-stabilized zirconium (Ziterion) | 0 | 3/13 | 3/13 |
| Cionca et al 2021(Cionca et al. 2021) | 46 | 29 | 63.04% | technical complication (aseptic loosening, n=5; fracture, n=8; primary failure, n=1);  biological complication (peri-implantitis, n=3); | Monolithic | two-piece zirconia implants (ZERAMEXâ T Implant System) | 3 | 1/48 | 24/25 |
| Spies et al 2017(Spies et al. 2017) | 22 | 20 | 90.91% | technical complications (occlusal roughness, n = 1; ceramic fracture, n = 1) | Monolithic | one-piece zirconia implants (Metoxit AG) | 2 | 4/20 | 12/12 |
| Spies et al 2019(Spies et al. 2019) | 40 | 31 | 77.50% | technical complications (major chippings, n = 5; increased occlusal roughness, n = 4) | Veneered | one‐piece yttria‐stabilized zirconia implants (VITA Zahnfabrik) | 0 | 0/40 | NS |
| Spies et al 2015(Spies et al. 2015) | 57 | 33 | 57.89% | technical complication (chipping complications, n = 14) | Veneered | zirconia implants (Biocare®) | 9 | 6/60 | NS |
| Spies et al 2015(Spies et al. 2015) | 52 | 20 | 38.46% | technical complication (chipping complications, n = 32) | FDP | zirconia implants (Biocare®) | 4 | 6/50 | NS |
| Spies et al 2018(Spies et al. 2018) | 26 | 12 | 46.15% | technical complication (occlusal roughness, n = 14) | FDP | One-piece ceramic implants (Metoxit AG) | 0 | 0/26 | NS |
| Spies et al 2019(Spies et al. 2019) | 22 | 20 | 90.91% | technical complication (major chippings, n = 2) | FDP | one‐piece yttria‐stabilized zirconia implants (VITA Zahnfabrik) | 0 | NS | NS |
| Total | 281 | 179 | 63.70% | - | - |  |  |  |  |
