# Supplementary material for: Clinical performance of zirconium implants compared to titanium implants: a systematic review and meta-analysis of randomized controlled trials
Source: PeerJ. 2023 Mar 17;11:e15010. doi: 10.7717/peerj.15010 (PMC10026713; doi:10.7717/peerj.15010)
Supplement: Table S4 [file peerj-11-15010-s009.docx]

**Table S4:**

Survival rate and reasons for implant failure in Zirconium implants.

| Study | Total | Success | Success rate | Reasons of prothesis failure | Type of prostheses | Implant | Drop out | Anterior/  Posterior | Maxilla/ Mandible |
| --- | --- | --- | --- | --- | --- | --- | --- | --- | --- |
| 1-year follow-up | | | | | |  |  |  |  |
| Henao et al 2021(Ruiz Henao et al. 2021) | 16 | 16 | 100.00% | - | Monolithic | ZLA Ceramic monotype implant (Straumann) | 0 | 16/0 | NS |
| Koller et al 2020(Koller et al. 2020) | 16 | 15 | 93.75% | technical complication (crown delivery, n = 1) | Monolithic | yttria-stabilized zirconium (Ziterion) | 0 | 3/13 | 3/13 |
| Osman et al 2014(Osman et al. 2014b) | 73 | 52 | 71.23% | technical complication (failed to osseointegrate, n = 18; implants fractured, n = 3) | Overdentures | Zirconium (Southern Implants) | 11 | 36/48 | 48/36 |
| Becker et al 2017(Becker et al. 2017) | 68 | 66 | 97.05% | technical complications (implant lost, n = 2) | Monolithic | zirconium implant system (Zircon Vision GmbH) | 0 | 0/68 | 18/50 |
| Cionca et al 2015(Cionca et al. 2015) | 46 | 41 | 89.13% | technical complication (aseptic loosening, n = 5) | Monolithic | two-piece zirconia implants (ZERAMEXâ T Implant System) | 3 | 1/48 | 24/25 |
| Cannizzaro et al 2010(Cannizzaro et al. 2010) | 40 | 35 | 87.50% | technical complication (implants failed, n = 5) | Monolithic | one-piece Z-Look3 zirconia implants (Z-Systems, Oensingen) | 0 | 18/22 | 29/11 |
| Spies et al 2017(Spies et al. 2017) | 24 | 24 | 100.00% | - | Monolithic | one-piece zirconia implants (Metoxit AG) | 0 | 4/20 | 12/12 |
| Borgonovo et al 2012(Borgonovo et al. 2012) | 20 | 20 | 100.00% | - | Monolithic | yttrium-stabilized zirconia (White-SKY® Bredent) | 9 | 10/10 | 14/6 |
| Kohal et al 2012(Kohal et al. 2012) | 62 | 59 | 95.4% | technical complications (implant failed, n=3) | Monolithic | yttria-stabilized zirconia (Nobel Biocare) | 4 | 6/50 | 14/42 |
| Spies et al 2015(Spies et al. 2015) | 62 | 62 | 100.00% | - | Veneered | zirconia implants (Biocare®) | 4 | 6/60 | NS |
| Jung et al 2015(Jung et al. 2016) | 49 | 48 |  | technical complications (implant lost, n=1) | Veneered | zirconia implants (VITA Zahnfabrik) | 0 | 3/68 | 23/48 |
| Kniha et al 2017(Kniha et al. 2018) | 105 | 105 |  | - | monotype | one-piece zirconia implants (Institut Straumann AG) | 12 | 64/41 (available at end of follow-up) | 82/23 |
| Spies et al 2019(Spies et al. 2019) | 44 | 44 | 100.00% | - | Veneered | one‐piece yttria‐stabilized zirconia implants (VITA Zahnfabrik) | 0 | 0/44 | 18/26 |
| Spies et al 2015(Spies et al. 2015) | 54 | 54 | 100.00% | - | FDP | zirconia implants (Biocare®) | 2 | 6/50 | NS |
| Jung et al 2015(Jung et al. 2016) | 22 | 22 | 100.00% | - | FDP | zirconia implants (VITA Zahnfabrik) | 0 | 3/68 | 23/48 |
| Spies et al 2018(Spies et al. 2018) | 24 | 24 | 100.00% | - | FDP | One-piece ceramic implants (Metoxit AG) | 2 | 0/26 | NS |
| Spies et al 2019(Spies et al. 2019) | 20 | 20 | 100.00% | - | FDP | one‐piece yttria‐stabilized zirconia implants (VITA Zahnfabrik) | 0 | NS | NS |
| Total | 745 | 707 | 94.90% | - | - |  |  |  |  |
| 2-year follow-up | | | | | |  |  |  |  |
| Michael Payer et al 2015(Payer et al. 2015) | 16 | 15 | 93.75% | technical complication (crown delivery, n = 1) | Monolithic | yttria-stabilized zirconium (Ziterion) | 0 | 3/13 | 3/13 |
| Koller et al 2020(Koller et al. 2020) | 16 | 15 | 93.75% | technical complication (crown delivery, n = 1) | Monolithic | yttria-stabilized zirconium (Ziterion) | 0 | 3/13 | 3/13 |
| Spies et al 2017(Spies et al. 2017) | 23 | 22 | 95.65% | technical complication (occlusal roughness, n = 1) | Monolithic | one-piece zirconia implants (Metoxit AG) | 1 | 4/20 | 12/12 |
| Becker et al 2017(Becker et al. 2017) | 68 | 66 | 93.75% | technical complications (implant lost, n = 2) | Monolithic | zirconium implant system (Zircon Vision GmbH) | 0 | 0/68 | 18/50 |
| Borgonovo et al 2012(Borgonovo et al. 2012) | 20 | 20 | 100.00% | - | Monolithic | yttrium-stabilized zirconia (White-SKY® Bredent) | 9 | 10/10 | 14/6 |
| Spies et al 2019(Spies et al. 2019) | 42 | 42 | 100.00% | - | Veneered | one‐piece yttria‐stabilized zirconia implants (VITA Zahnfabrik) | 2 | 0/44 | 18/26 |
| Spies et al 2015(Spies et al. 2015) | 62 | 62 | 100.00% | - | Veneered | zirconia implants (Biocare®) | 4 | 6/60 | NS |
| Spies et al 2015(Spies et al. 2015) | 50 | 50 | 100.00% | - | FDP | zirconia implants (Biocare®) | 6 | 6/50 | NS |
| Spies et al 2018(Spies et al. 2018) | 24 | 24 | 100.00% | - | FDP | One-piece ceramic implants (Metoxit AG) | 2 | 0/26 | NS |
| Total | 321 | 316 | 98.44% | - | - |  |  |  |  |
| 5-year follow-up | | | | | |  |  |  |  |
| Koller et al 2020(Koller et al. 2020) | 16 | 14 | 87.50% | technical complications (crown delivery, n = 2) | Monolithic | yttria-stabilized zirconium (Ziterion) | 0 | 3/13 | 3/13 |
| Cionca et al 2021(Cionca et al. 2021) | 46 | 38 |  | technical complication (aseptic loosening, n=5; mechanical failure, n=1; primary failure, n=1);  biological complication (peri-implantitis, n=1); | Monolithic | two-piece zirconia implants (ZERAMEXâ T Implant System) | 3 | 1/48 | 24/25 |
| Spies et al 2017(Spies et al. 2017) | 22 | 22 | 100.00% | - | Monolithic | one-piece zirconia implants (Metoxit AG) | 2 | 4/20 | 12/12 |
| Borgonovo et al 2012(Borgonovo et al. 2012) | 20 | 20 | 100.00% | - | Monolithic | yttrium-stabilized zirconia (White-SKY® Bredent) | 9 | 10/10 | 14/6 |
| Spies et al 2019(Spies et al. 2019) | 40 | 40 | 100.00% | - | Veneered | one‐piece yttria‐stabilized zirconia implants (VITA Zahnfabrik) | 0 | 0/40 | NS |
| Spies et al 2015(Spies et al. 2015) | 57 | 47 | 82.46% | technical complications (severity of the chipping, n = 10) | Veneered | zirconia implants (Biocare®) | 9 | 6/60 | NS |
| Spies et al 2015(Spies et al. 2015) | 52 | 32 | 61.53% | technical complications (severity of the chipping, n = 20) | FDP | zirconia implants (Biocare®) | 4 | 6/50 | NS |
| Spies et al 2018(Spies et al. 2018) | 26 | 26 | 100.00% | - | FDP | One-piece ceramic implants (Metoxit AG) | 0 | 0/26 | NS |
| Spies et al 2019(Spies et al. 2019) | 22 | 22 | 100.00% | - | FDP | one‐piece yttria‐stabilized zirconia implants (VITA Zahnfabrik) | 0 | NS | NS |
| Total | 301 | 261 | 86.71% | - | - |  |  |  |  |
