# Supplementary material for: Clinical performance of zirconium implants compared to titanium implants: a systematic review and meta-analysis of randomized controlled trials
Source: PeerJ. 2023 Mar 17;11:e15010. doi: 10.7717/peerj.15010 (PMC10026713; doi:10.7717/peerj.15010)
Supplement: Table S5 [file peerj-11-15010-s010.docx]

**Table S5:**

Meta-regression on survival rate and success rate of zirconia implants.

| Covariates | Survival rate | | | | |  | Success rate | | | | |
| --- | --- | --- | --- | --- | --- | --- | --- | --- | --- | --- | --- |
|  | Coef | z | *P* | 95% CI | |  | Coef | z | *P* | 95% CI | |
|  |  |  |  | Lower | Upper |  |  |  |  | Lower | Upper |
| SC% | 0.010 | 0.27 | 0.784 | -0.068 | 0.094 |  | 0.175 | 2.02 | 0.043 | 0.005 | 0.345 |
| Anterior% | -0.047 | -0.66 | 0.510 | -0.266 | 0.096 |  | -0.023 | -0.08 | 0.933 | -0.559 | 0.513 |
| Mandible% | -0.015 | -0.23 | 0.818 | -0.217 | 0.123 |  | -0.207 | -1.13 | 0.257 | -0.564 | 0.150 |
| follow-up | -0.005 | -0.59 | 0.555 | -0.021 | 0.012 |  | -0.038 | -1.94 | 0.052 | -0.077 | 0.000 |

**Abbreviations:** Coef, coefficient; CI, confidence intervals.
