# Supplementary material for: Clinical performance of zirconium implants compared to titanium implants: a systematic review and meta-analysis of randomized controlled trials
Source: PeerJ. 2023 Mar 17;11:e15010. doi: 10.7717/peerj.15010 (PMC10026713; doi:10.7717/peerj.15010)
Supplement: Supplemental Information 2 [file peerj-11-15010-s011.docx]

**For systematic reviews / meta-analyses, authors need to provide the following information:**

**1. The rationale for conducting the systematic review / meta-analysis;**

**Reply:**

**(1) Whether Zr implants could be widely used in clinical practices remained controversial among preclinical and clinical evidence.**

**(2) The previous systematic review could not provide updated evidence based on RCTs, which are of the highest evidence level.**

**2. The contribution that it makes to knowledge in light of previously published related reports, including other meta-analyses and systematic reviews**

**Reply:**

**There are two main contributions.**

**(1)** Several systematic reviews and meta-analyses have compared the properties of Zr and Ti implants. A systematic review of in vivo experiments (published in 2014) found no difference in bone-implant contact (BIC) and removal torque between Zr and Ti implants. (Manzano et al. 2014) Another meta-analysis published in 2017 did not compare Zr and Ti implants in the randomized controlled trials (RCTs) with the comparable baseline and has been out of date. (Elnayef et al. 2017) The recent systematic review did not quantitively analyze the difference between Zr and Ti implants.(Fernandes et al. 2022) **Our systematic review and meta-analysis of RCTs will provide up-to-date evidence and recommendations.**

**(2)** We found that the RCTs on this topic is limited. Then, in the discussion, we further collected and analyzed the survival rate, success rate, failure reasons in observational studies. **Our paper will provide not only up-to-date evidence based on RCTs but also a comprehensive review based on all the comparative studies of Ti vs. Zr implants.**
